# Diagnosing enterovirus meningitis via blood transcriptomics: an alternative for lumbar puncture?

**DOI:** 10.1186/s12967-019-2037-6

**Published:** 2019-08-23

**Authors:** Esther Bartholomeus, Nicolas De Neuter, Annelies Lemay, Luc Pattyn, David Tuerlinckx, David Weynants, Koen Van Lede, Gerlant van Berlaer, Dominique Bulckaert, Tine Boiy, Ann Vander Auwera, Marc Raes, Dimitri Van der Linden, Helene Verhelst, Susanne Van Steijn, Tijl Jonckheer, Joke Dehoorne, Rik Joos, Hilde Jansens, Arvid Suls, Pierre Van Damme, Kris Laukens, Geert Mortier, Pieter Meysman, Benson Ogunjimi

**Affiliations:** 1 Center of Medical Genetics, University of Antwerp/Antwerp University Hospital, Edegem, Belgium; 2 AUDACIS, Antwerp Unit for Data Analysis and Computation in Immunology and Sequencing, University of Antwerp, Antwerp, Belgium; 3 Adrem Data Lab, Department of Mathematics and Computer Science, University of Antwerp, Antwerp, Belgium; 4 Biomedical Informatics Research Network Antwerp (Biomina), University of Antwerp, Antwerp, Belgium; 5 Department of Paediatrics, AZ Turnhout, Turnhout, Belgium; 6 Université Catholique de Louvain/CHU UCL Namur, Site Dinant, Service de Pédiatrie, Dinant, Belgium; 7 Department of Paediatrics, CHU ULC Namur Ste Elisabeth, Namur, Belgium; 8 Department of Paediatrics, AZ Nikolaas, Sint-Niklaas, Belgium; 9 Department of Emergency Medicine/Pediatric Care, University Hospital Brussels, Jette, Belgium; 10 Department of Paediatrics, Antwerp University Hospital, Edegem, Belgium; 11 Department of Paediatrics, GZA Sint-Augustinus, Wilrijk, Belgium; 12 Department of Paediatrics, Jessa Hospital, Hasselt, Belgium; 13 Paediatric Infectious Diseases, Department of Paediatrics, CHU ULC Cliniques Universitaires Saint-Luc, UCLouvain, Brussels, Belgium; 14 Department of Paediatric Rheumatology, University Hospital, Ghent, Belgium; 15 Department of Paediatrics, ZNA Paola, Antwerp, Belgium; 16 Department of Paediatrics, GZA Sint-Vincentius, Antwerp, Belgium; 17 Antwerp Center for Paediatric Rheumatology and AutoInflammatory Diseases, Antwerp, Belgium; 18 Department of Laboratory Medicine, Antwerp University Hospital, Edegem, Belgium; 19 Centre for the Evaluation of Vaccination (CEV), Vaccine & Infectious Disease Institute (VAXINFECTIO), University of Antwerp, Antwerp, Belgium; 20 Centre for Health Economics Research & Modeling Infectious Diseases (CHERMID), Vaccine & Infectious Disease Institute (VAXINFECTIO), University of Antwerp, Antwerp, Belgium; 21 Antwerp Center for Translational Immunology and Virology (ACTIV), Vaccine & Infectious Disease Institute (VAXINFECTIO), University of Antwerp, Universiteitsplein 1, 2610 Wilrijk, 00323/8213251 Antwerp, Belgium; 22 Department of Pediatrics, University Hospital Brussels, Jette, Belgium

**Keywords:** Meningitis, Enterovirus, Bacterial meningitis, Differential gene expression

## Abstract

**Background:**

Meningitis can be caused by several viruses and bacteria. Identifying the causative pathogen as quickly as possible is crucial to initiate the most optimal therapy, as acute bacterial meningitis is associated with a significant morbidity and mortality. Bacterial meningitis requires antibiotics, as opposed to enteroviral meningitis, which only requires supportive therapy. Clinical presentation is usually not sufficient to differentiate between viral and bacterial meningitis, thereby necessitating cerebrospinal fluid (CSF) analysis by PCR and/or time-consuming bacterial cultures. However, collecting CSF in children is not always feasible and a rather invasive procedure.

**Methods:**

In 12 Belgian hospitals, we obtained acute blood samples from children with signs of meningitis (49 viral and 7 bacterial cases) (aged between 3 months and 16 years). After pathogen confirmation on CSF, the patient was asked to give a convalescent sample after recovery. 3′ mRNA sequencing was performed to determine differentially expressed genes (DEGs) to create a host transcriptomic profile.

**Results:**

Enteroviral meningitis cases displayed the largest upregulated fold change enrichment in type I interferon production, response and signaling pathways. Patients with bacterial meningitis showed a significant upregulation of genes related to macrophage and neutrophil activation. We found several significantly DEGs between enteroviral and bacterial meningitis. Random forest classification showed that we were able to differentiate enteroviral from bacterial meningitis with an AUC of 0.982 on held-out samples.

**Conclusions:**

Enteroviral meningitis has an innate immunity signature with type 1 interferons as key players. Our classifier, based on blood host transcriptomic profiles of different meningitis cases, is a possible strong alternative for diagnosing enteroviral meningitis.

## Background

Patients presenting with meningitis need accurate and fast diagnosis to determine the adequate treatment. Meningitis is mainly caused by viral and bacterial infections, with enteroviruses as most common pathogen in children [1, 2]. Enteroviral meningitis (EVM) normally does not necessitate specific therapy except supportive therapy. In contrast, bacterial meningitis (BM), beyond neonatal period mainly caused by strains of *Neisseria meningitidis*, *Streptococcus pneumoniae* or rarely *Haemophilus influenzae*, poses a very severe situation with a high morbidity and mortality rate [3]. As physical symptoms might not be sufficient enough to distinguish between viral and bacterial meningitis, blood and more importantly cerebrospinal fluid (CSF) are used to identify the causative pathogen. Moreover, several clinical tools have been developed in order to assist the clinical decision-making process [4–7]. Multiplex PCR techniques on blood and CSF have been able to identify genes from pathogens, but achieving high sensitivity and specificity still pose a challenge nowadays. As such, bacterial culture still remains the golden standard [8]. However, bacterial culture can take up to 72 h before providing conclusive results for organisms that are difficult to grow or when the patient is partially treated with antimicrobial agents. Given the risk of delayed antibiotic administration in cases of bacterial meningitis, broad-spectrum antibiotics are used as soon as the clinical diagnosis of meningitis is made despite the lack of pathogenic identification, thereby potentially leading to an overuse of antibiotics and anti-microbial resistance. Importantly, the requirement of obtaining CSF through lumbar puncture for accurate diagnostics is not always feasible due to risk of brain herniation, hemodynamic instability or bleeding problems.

The pitfalls in pathogen-based diagnostics and the need to collect CSF could be (partially) mitigated by performing deep profiling of the host’s immune response to specific pathogens in blood. Several studies have used cytokine profiling as a method to differentiate between bacterial and viral meningitis, and although some usability was noted, the specificity and sensitivity remained too low [9, 10]. A more promising technique could be gene expression profiling of whole blood. Previous studies showed that, based on microarrays, differentially expressed genes (DEGs) pathways can be used to discriminate between different types of infection [3, 8, 11–19]. Different influenza strains and respiratory syncytial virus were studied by Herberg et al., Mejias et al. and Woods et al. in [14, 15, 17]. Other studies included meningitis cases to their bacterial or viral infection groups [8, 11–13]. Only the study of Lill et al., focused on a specific bacterial meningitis signature using microarrays [3]. However, this study included mainly neonates and adults. Together these studies indicate that viral and bacterial infections trigger different immune pathways of the host.

In this study, focusing only on bacterial and viral meningitis cases, we aimed to identify a unique enteroviral DEG signature in children presenting with meningitis using 3′ mRNA sequencing. Next, we assessed whether it was possible to distinguish bacterial from viral meningitis based on specific up- and downregulated pathways.

## Methods

### Study cohort

Twelve Belgian hospitals recruited patients for this multicentric prospective study from December 2015 until November 2017. Paediatricians and emergency physicians collected PaxGene blood RNA tubes (PreAnalytiX) samples from children, aged 3 months to 16 years, with symptoms reminiscent of meningitis. An acute sample was taken during an early diagnostic blood draw. After receiving written consent and confirmation of meningitis (identifying the causative pathogen by PCR or bacterial culture analysis of the CSF), patients were included in this study. After recovering, patients were asked to give a convalescent blood sample 1 to 3 months after remission. As this second sample was not obligatory, not all patients or their parents agreed to a second blood draw.

We collected 49 enteroviral meningitis (EVM) samples (with [EVM1] or without [EVM2] convalescence samples), 2 other (herpes simplex virus and varicella-zoster virus) viral samples (VM) and 7 bacterial meningitis samples (BM). Six BM samples are affected by a bacterial strain (BM1). The seventh BM sample (BM2) came from a patient with neuroborreliosis. As this is a rare case, not all analyses took this BM2 sample into account. Table 1 shows all samples per meningitis type and whether or not a convalescent sample was available. In order to increase the specificity of the EVM-specific signature, we also recruited 14 patients with known rheumatologically conditions as non-infectious inflammatory background samples (Additional file 1: Diagnoses).

**Table 1:** Overview of the different diagnosis groups

Diagnosis (incl./excl. convalescence sample)	Number of samples	Group abbreviation
Viral meningitis samples		
Enterovirus incl. convalescence sample	35	EVM1
Enterovirus excl. convalescence sample	12	EVM2
Herpes simplex type I excl. convalescence sample	1	VM
Varicella excl. convalescence sample	1	VM
Bacterial meningitis samples		
*Neisseria meningitidis* B excl. convalescence sample	2	BM1
*Neisseria meningitidis* B incl. convalescence sample	1	BM1
*Streptococcus pneumoniae* excl. convalescence sample	2	BM1
*Haemophilus influenza* incl. convalescence sample	1	BM1
Neuroborreliose bact. *Borrelia burgdorferi* exl. convalescence sample	1	BM2
Non-infectious inflammatory background samples		
Paediatric rheumatological conditions	14	REU

### RNA extraction

RNA extraction from PaxGene tubes was performed via a column-based RNA extraction using the PaxGene blood RNA extraction kit (Qiagen). To optimize RNA concentrations, we used the RNA clean & concentrator-5 kit (Zymo research). We verified the RNA quality using the Experion (Biorad, Experion RNA StdSens Analysis Kit). No RNA samples had to be excluded based on low quantity or quality.

### 3′ mRNA sequencing library prep and sequencing

All RNA samples were prepared with the QuantSeq3′ mRNA-Seq Library Prep Kit FWD for Illumina (Lexogen GmbH) following the standard supplier’s protocol for long fragments [20]. During the RNA removal step we also added globin blockers, so none of the abundant globin mRNA was copied to double stranded cDNA. The resulting cDNA libraries were equimolarly pooled, with up to 40 samples for one NextSeq 500 sequencing run (high output v2 kit, 150 cycli, single read, Illumina). This gave us an optimum of 10 million reads for each sample.

### Data analysis and statistics

The sequencing data is available in the Gene Expression Omnibus (GSE133378). All codes used for the preprocessing and analysis of the data within this manuscript as well as the codes used to generate results is publicly available on github (https://github.com/NDeNeuter/GEMS).

#### NGS data processing

Raw data from the NextSeq was demultiplexed and further processed through an in-house developed 3′mRNA sequencing pipeline. The quality of all reads was evaluated using FastQC (v0.11.5) before and after processing with Trimmomatic (v0.36) [21, 22]. Trimmomatic removed the leading 20 bases from reads, ensure a minimum quality score of 15 over a sliding window of 4 bases and require a minimum read length of 30 bases. Usage of oligodT primers could cause poly-A stretches at the 3′ end. To remove these poly-A stretches, the 3′ read end was trimmed with our own in-house poly-A removal script. All sequences remaining after trimming were mapped against the human reference genome build 38 (polymorph variants excluded) with HISAT2 (v2.0.4) [23]. HTseq (v0.6.1) was used to count all reads for each gene and set up a read count table [24].

#### Differential gene expression analysis and gene ontology enrichment analysis

Differential gene expression analyses were performed using the DESeq 2 Bioconductor package [25]. For any given differential gene expression analysis, genes with less than 300 read counts over all samples considered during the analysis were removed prior to the analysis. Gene ontology enrichment analysis was performed on significantly differentially expressed genes with a log2 fold change of at least 1 (either up- or downregulated) using PANTHER’s online gene ontology enrichment tool (gene ontology version: 1.2, gene ontology annotations: 2018-10-08) [26]. To determine significantly enriched/depleted gene ontology terms relating to biological processes, a Fisher’s exact test was performed with Bonferroni correction for multiple testing. As reference background set of genes, we used the 21,721 genes measured during the 3′-mRNAseq experiment.

#### Random forest with feature selection classifier

Classification of samples was performed by Random Forest classifiers as implemented in Scikit-Learn. Classifiers were initialized using 1000 estimators and balanced class weights, leaving other parameters at their default values [27]. The random forest classifier was trained on the normalized gene expression values for each measured gene as features. Due to the larger number of genes available to the model, a feature selection step was used. The feature selection step used the Boruta method with a Bonferroni correction to only retain informative genes/features [28]. The Bonferroni correction was set to either the default parameter (α = 0.05) or to the more strict threshold (α = 0.001). Validation of classifiers was performed using a leave-one-out cross validation strategy. In this strategy, a single sample is removed from the set of samples under consideration. Detection of differentially expressed genes and feature selection is applied to the remaining samples. The selected features are then used to train a classification model which is tested on the left-out sample. This approach is repeated for each sample under consideration to obtain an overall indication of performance. To evaluate performance, receiver-operator-characteristic (ROC) curves were drafted and the area under the ROC curve (AUC) was calculated.

### Ethical forms

This non-commercial study was approved by the IRBs from the Antwerp University Hospital/University of Antwerp and all local committees from each hospital (EC-15/43/448, Belgian registration number: B300201526554).

## Results

### Enteroviral meningitis-specific transcriptomic profile

We compared the transcriptomic profile of acute enteroviral meningitis samples from 35 patients to their own convalescent sample to identify pathways altered due to the enteroviral infection (EVM1 group). 2380 DEGs (2108 upregulated and 272 downregulated genes) were found between acute and convalescence samples (Additional file 2). For further interpretation, the resulting differentially expressed genes (DEGs) obtained after a differential gene expression analysis were translated to corresponding Gene Ontology (GO) categories with a minimum log2 fold enrichment of 1, thus at least twofold log change (Additional file 3). Interestingly, the GO analysis showed a predominant upregulation of the type I interferon (IFN) signature (Additional file 3) with three type I IFN related GO categories within the top 4 GO terms with a fold enrichment value of 5.09 to 5.60: (1) type I interferon signaling pathway (5.60), (2) cellular response to type I interferon (5.60), and (3) response to type I interferon (5.09). The 10 strongest IFN-related single DEGs are (in decreasing order): IFIT1, IFI44L, RSAD2, OAS3, OASL, MX1, IFIT3, EIF2AK2, IFITM3 and IFI44 (Additional file 2). Furthermore, the GO analysis showed that enteroviral meningitis might affect the negative regulation of viral genome replication, protein targeting to ER, the detection and response to foreign virus particles and many more biological and metabolic processes (Additional file 3).

### Bacterial meningitis-specific transcriptomic profile

Six patients in our cohort were diagnosed with bacterial meningitis (BM1), not taking into account the case of neuroborreliosis (BM2)(Table 1). For two of these BM1 patients, we were able to obtain a second convalescent sample. Given that only one significant differential expressed gene, namely the pseudogene FTH1P11, was found between the bacterial convalescent and enteroviral convalescent samples (Additional file 4: Figure S1), we proceeded using two methods: (A) a paired comparison between the two samples for which both a bacterial meningitis and convalescent sample were available and (B) an unpaired comparison between the six bacterial meningitis samples and all convalescent samples (originating from patients with either enteroviral or bacterial meningitis). We only retained the DEGs and GO categories for results that were found in both methods.

#### Method A: BM1 longitudinal DEG analysis

We found GO categories related to innate immunity including macrophage activation (Top 1 category; fold enrichment of 9.16), and mainly granulocytes activation and degranulation, in especially neutrophil activation (regulation IL-8 = neutrophil-activating factor (NAF) production) with neutrophilic marker CD177 as strongest single DEG. As expected, we noticed the detection of DEGs involved in bacterial lipopeptides and –proteins (and cellular response against it) as well. Furthermore, we found signs of T cell immunity with T cell activation in immune response (2nd GO, 7.04), T cell differentiation (20th GO, 5.25), T cell activation (32nd GO, 4.55) and three more T cell regulation GO terms with a lower-fold enrichment. In addition, we found GO categories with a lower-fold enrichment that are related to general cytokine production and regulation, inflammatory responses and more general (myeloid) leukocyte/lymphocyte related regulation, differentiation and activation. The lowest fold enrichments are reserved for more non-immune general pathways as signaling, metabolic reactions and enzyme/transcription factor activities (Additional file 5 and 6).

#### Method B: acute BM1 vs. pooled convalescence samples

**Fig. 1:**
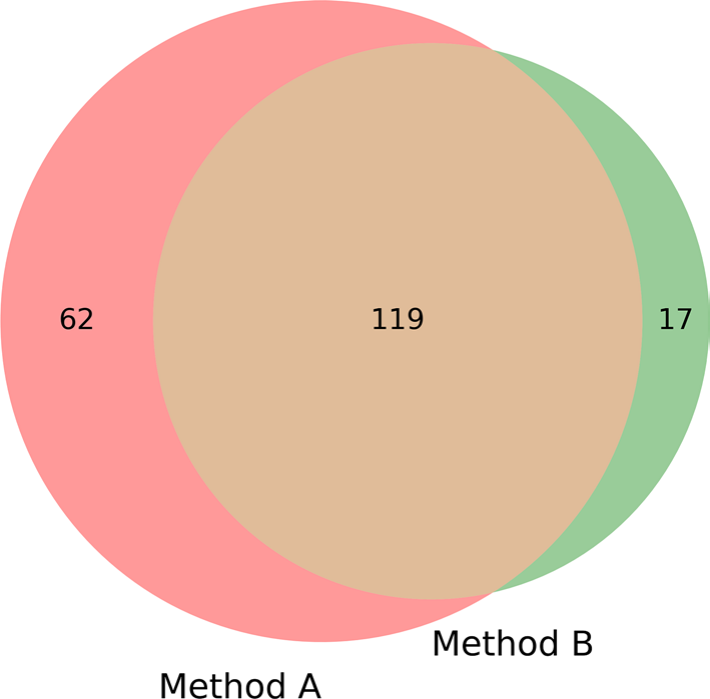
Overlap GO terms of both BM analysis. Venn-diagram showing the overlap of GO terms between method A and method B of the bacterial meningitis-specific transcriptomic profile

Top responses in this analysis were similar to those found in the BM1 longitudinal analysis: innate immunity related GO categories such as granulocyte activation and degranulation and the more general (myeloid) leukocyte/lymphocyte categories and the T cell related GOs (Fig. 1). Also, single DEGs were quite similar as for Method A (Additional files 7 and 8).

#### Summarized trends in both Method A and B analyses

A clear trend could be seen within both comparisons between bacterial and convalescent samples towards an activation of the innate immune system, in particular macrophages and neutrophils, and a sign of T cell activation was noted for patients with bacterial meningitis.

### Gene expression differences between enteroviral and bacterial meningitis

680 DEGs (349 downregulated and 331 upregulated genes) could be identified in 47 acute EVM1/EVM2 samples compared to the six BM1 samples (Additional file 9). Based on all measured DEGs, the most significant GO term is the regulation of tumor necrosis factor (TNF) secretion with a fold enrichment of 11.68. This is directly followed by an upregulation of three type I IFN related terms in EVM patients compared to BM patients [response to type I IFN (10.90; 22 DEGs), type I IFN signaling pathway (10.88; 20 DEGs) and cellular response to type I IFN (10.88; 20 DEGs), all DEGs upregulated with positive log2fold changes], which was also found in the longitudinal EVM analysis (Additional files 2 and 10). Furthermore, we noted other viral related GO terms concerning viral life cycle, replication and regulation with a high fold enrichment, as expected (Additional file 10), followed by more general protein localization and targeting GO terms. In the lower GO terms with a fold enrichment below 3.5 neutrophilic and leukocyte responses appear, together with cytokines regulation and production. These lower GO terms were also found in the longitudinal BM analysis (Additional files 5 and 7). However here the associated single DEGs have a negative log2fold change, meaning that they are downregulated in the EVM samples compared to the upregulation in BM samples (Additional file 10). To study the DEGs in more detail, we performed a separate GO analysis on the 331 upregulated and the 349 downregulated DEGs (Additional file 10). As expected, the upregulated DEGs leaded to four immune-related GOs: defense response to virus, regulation of multi-organism process, immune effector process and immune system process. The downregulated DEGs were traced to 12 different GO terms, where only the last two terms were immune-related: inflammatory response and defense response (Additional file 11).

A similar analysis was performed using all enteroviral and bacterial samples (including BM2), which is discussed in Additional file 12: Results.

### Enteroviral versus bacterial meningitis classifier

**Fig. 2:**
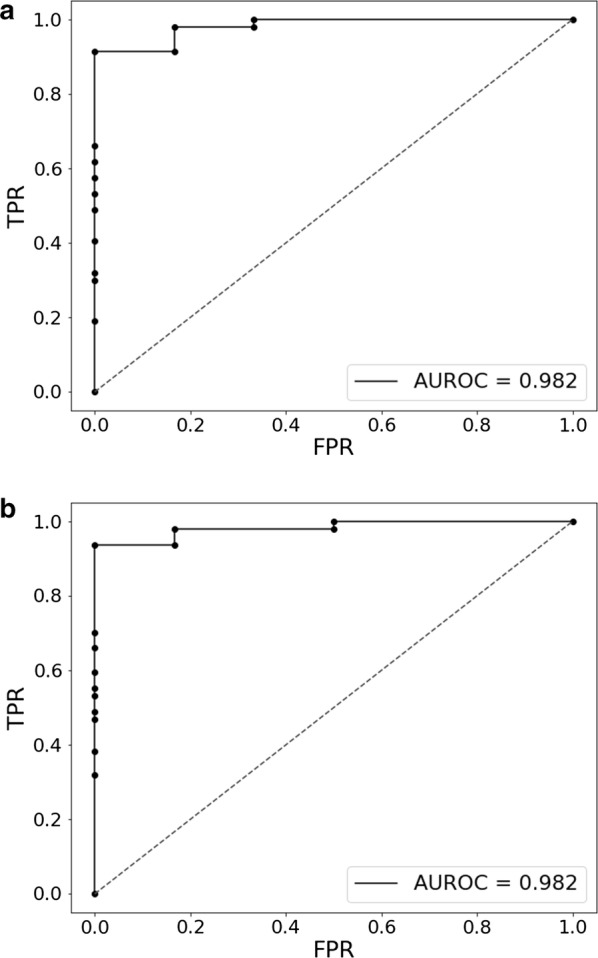
**a** EVM1/2-BM1 classifier ROC curve. Random forest classifier between EVM1/2 and BM1 (including feature selection and Bonferroni correction, α = 0.05) resulted in 56 classifier genes. **b** EVM1/2-BM1 classifier ROC curve. Random forest classifier between EVM1/2 and BM1 (including feature selection and Bonferroni correction, α = 0.001) resulted in 37 classifier genes

In the last step we used the normalized gene expression values from the EVM1/2 versus BM1 samples to build a random forest classifier that would be able to distinguish enteroviral meningitis cases from acute bacterial cases. After cross-validation, we obtained an AUC value of 0.982 (Fig. 2a), indicating that the classifier is able to discriminate enteroviral from bacterial samples. To determine which genes were indicative of the meningitis type of the sample, the genes that passed the feature selection step (Bonferroni α = 0.05) were extracted from a random forest model that was trained on all the EVM and BM samples. In total, a set of 56 predictive genes were identified in this way (Additional file 13). To assess whether a smaller set of genes could be equally or comparably performant, the feature selection step was made stricter (Bonferroni α = 0.001). This stricter method identified a predictive gene set of 37 genes with an AUC of 0.982 (Fig. 2b and Additional file 13, Additional file 14: Figure S2). Most of the 56 and 37 predictive classifier genes are present as DEG, found in the EVM1/EVM2 versus BM1 analysis (Additional file 13).

**Fig. 3:**
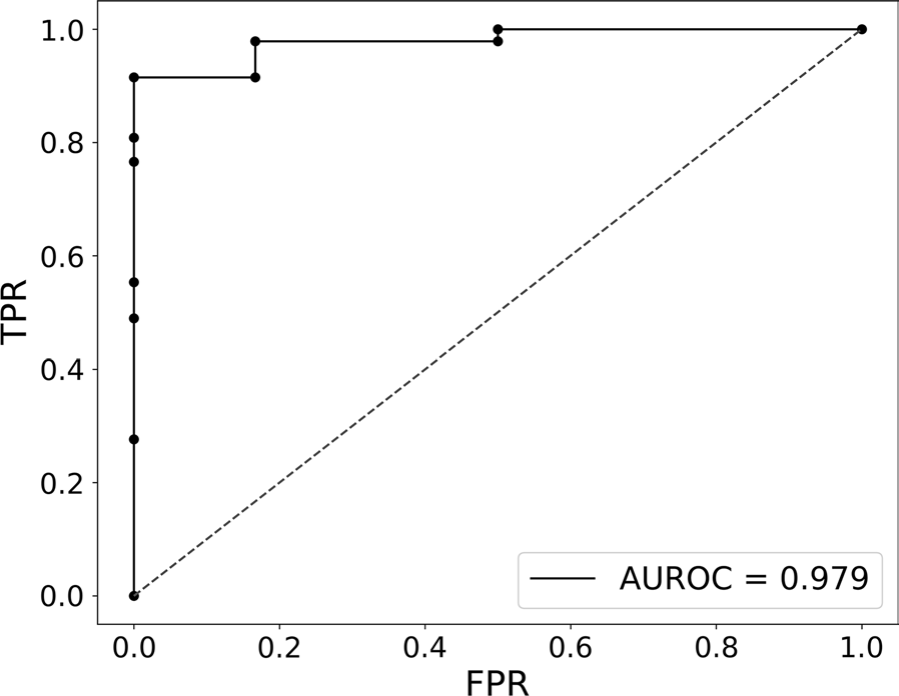
Application of literature classifier on resulting dataset (ROC curve). Classifier performance to distinguish our enteroviral samples from bacterial samples, based on 41 genes, selected from a literature bacterial-viral infection classifier

In addition, we gathered a set of 41 genes that had previously been implicated with viral versus bacterial infections from a recently published “general” classifier from Herberg et al., 2016 [13]. Using the same leave-one-out cross validation strategy, we tested how well this set of 41 genes was able to predict whether an unknown sample was diagnosed as enteroviral or bacterial meningitis. We obtained an AUC value of 0.979 (Fig. 3), which shows that the two sets of signature genes are equally performant.

**Fig. 4:**
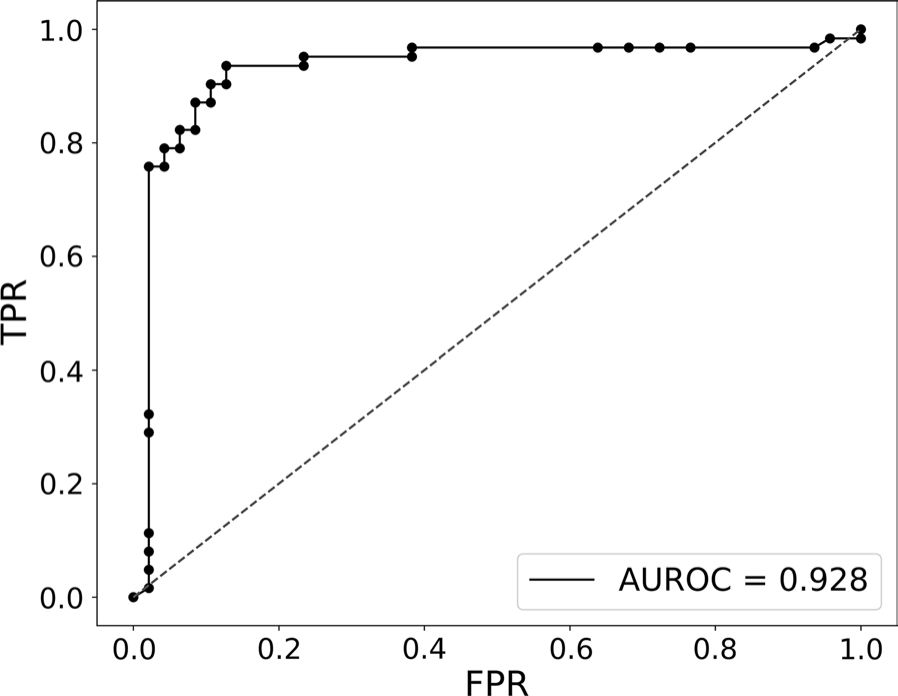
EVM specific classifier ROC curve. Enteroviral meningitis classifier select 61 genes to distinguish EVM from other types of infections

Finally, for potential clinical applications, we investigated whether it is possible to specifically identify enteroviral meningitis samples from any infectious sample. To attain this goal, we trained a classifier on all our viral meningitis samples versus all other samples (including background samples from paediatric patients with non-infectious inflammatory conditions (Additional file 1) and patients with other viral meningitis causes) following the same method as described for the enteroviral versus bacterial classifier. We obtained an AUC value of 0.928 (Fig. 4), indicating excellent performance, and identified a set of 61 genes that were predictive of the sample being an enteroviral meningitis sample (Additional file 15). Only five of those classifier genes are not present as DEG in the EVM1 versus control analysis (Additional file 15).

## Discussion

In this study, we showed for the first time that the analysis of 3′mRNA sequencing data from whole blood can adequately distinguish enteroviral meningitis from bacterial meningitis. Using our longitudinal data, we found that the upregulation of type I IFN genes was one of the most striking observations in enteroviral meningitis, whereas the activation of macrophages and neutrophils was a dominant feature in bacterial meningitis, similar to what has been noted before for viral and bacterial comparisons [3, 8, 11, 12]. Including rarer causes of meningitis, like *Borrelia burgdorferi* (BM2 sample), had no influence on the main type I IFN or neutrophilic patterns. The differences in gene expression between enteroviral and bacterial meningitis made it possible to build a classifier with an AUC of 0.975. We noted that the earlier published classifier genes (41 genes) between different types of viral and bacterial infections also led to similar AUC as ours, thereby supporting the use of both classifiers in practice [13]. Moreover, our results showed that with a reasonable AUC of 0.924 enteroviral meningitis could be differentiated from not only bacterial meningitis, but also herpes meningitis and non-infectious inflammatory rheumatologically conditions.

Our findings are not only of scientific interest for a better understanding of the underlying pathophysiology and the identification of potential therapeutic targets but might also—with the foreseen reduction of turnover time in sequencing in mind—present new possibilities in clinical practice when lumbar puncture is not feasible, delayed or when the direct identification of the pathogens is uncertain, due to negative cultures or PCR. In addition, during enterovirus season and given a unconfirmed clinical diagnosis of enteroviral meningitis, our findings suggest that our classifier is capable of verifying this clinical diagnosis, and thus potentially avoiding an unnecessary lumbar puncture as well as antibiotic administration.

Our study is, despite the significant results, limited by the sample size of each different bacterial strain. In addition, for most bacterial cases, we were unable to acquire a convalescent sample. To overcome these limitations, we grouped samples of the 3 most common bacterial strains (BM1) and all collected convalescence samples. With those 6 BM1 samples and all EVM samples we were able to create a performant general enteroviral-bacterial classifier. Including more samples could increase the discriminating strength of this classifier. In literature, there are classifiers available for other infections which can identify the specific strain of each infectious agent [3, 8, 11, 12]. Another caveat is that the use of high-throughput sequencing within a research setting is likely not feasible in routine clinical labs, due to the costs, the need of a library preparation and time limits. However, this may change as high-throughput sequencing becomes cheaper and faster in the future. More important, our findings revealed that a small set of genes were sufficient for classification, potentially enabling the development of a multiplex real-time PCR. Different primers could be designed, targeting the transcripts of all selected classifier genes. The input for the PCR requires total or mRNA, which is easily extracted from whole blood. Provided the needed optimization (primer design, input concentration and annealing temperature and duration), a real-time PCR is a fast technique and easy to integrate in routine clinical labs.

## Conclusions

Using 3′ mRNA sequencing, gene expression profiles can be composed for enteroviral and bacterial meningitis patients. Based on the DEGs we found the expected type I interferon signature for all enteroviral meningitis patients and we discovered a mainly neutrophilic pattern, supported by T-cell activation in bacterial meningitis patients. Even with a small number of bacterial meningitis samples, it is feasible to create a very well performing classifier that distinguish enteroviral meningitis cases from bacterial meningitis.

## Supplementary information

**Additional file 1.** Rheuma samples diagnosis.

**Additional file 2.** EVM1 vs C (DEGs fold1_sig).

**Additional file 3.** EVM1 vs C (GO terms).

**Additional file 4: Figure S1.** MA plot of all convalescence samples. MA plot shows the relationship of the log fold changes, plotted to the average of the normalized counts, between the convalescence samples of BM1 and EVM1 group. Each gene is represented with a dot and are colored red if the adjusted p-value is below 0.1.

**Additional file 5.** BM1 vs C (GO terms).

**Additional file 6.** BM1 vs C (DEGs fold1_sig).

**Additional file 7.** BM1 vs C_all (GO terms).

**Additional file 8.** BM1 vs C_all (DEGs fold1_sig).

**Additional file 9.** EVM1_EVM2 vs BM1 (DEGs fold1_sig).

**Additional file 10.** EVM1_EVM2 vs BM1 (GO terms).

**Additional file 11.** EVM1_EVM2 vs BM1_BM2 (GO terms).

**Additional file 12: Additional Results.** Gene expression differences between enteroviral and bacterial meningitis including the neuroborreliosis case.

**Additional file 13.** Classifier genes EVM vs BM1 (56 and 37 genes).

**Additional file 14: Figure S2.** Heatmap of the EVM1/2-BM1 classifier genes. Clustered heatmap of the resulting 37 genes (log transformed normalized read counts) of the EVM1/2 versus BM1 strict classifier [Ward’s linkage clustering with euclidean distances].

**Additional file 15.** Classifier genes EVM specific genes.

## Data Availability

All raw and processed data is available in the Gene Expression Omnibus: GSE133378. All resulting datasets analyzed during this study are included in this published article and its additional files. All codes used for the preprocessing and analysis of the data within this manuscript as well as the codes used to generate results is publicly available on github at https://github.com/NDeNeuter/GEMS.

## Abbreviations

AUC: area under the ROC curve
BM: bacterial meningitis
CSF: cerebrospinal fluid
DEGs: differentially expressed genes
EVM: enteroviral meningitis
GO: gene ontology
IFN: interferon
NAF: neutrophil-activating factor
ROC: receiver-operator-characteristic curve
VM: viral meningitis
