# Supplementary figures and images for: Diagnosing enterovirus meningitis via blood transcriptomics: an alternative for lumbar puncture?
Source: J Transl Med. 2019 Aug 23;17:282. doi: 10.1186/s12967-019-2037-6 (PMC6708255; doi:10.1186/s12967-019-2037-6)

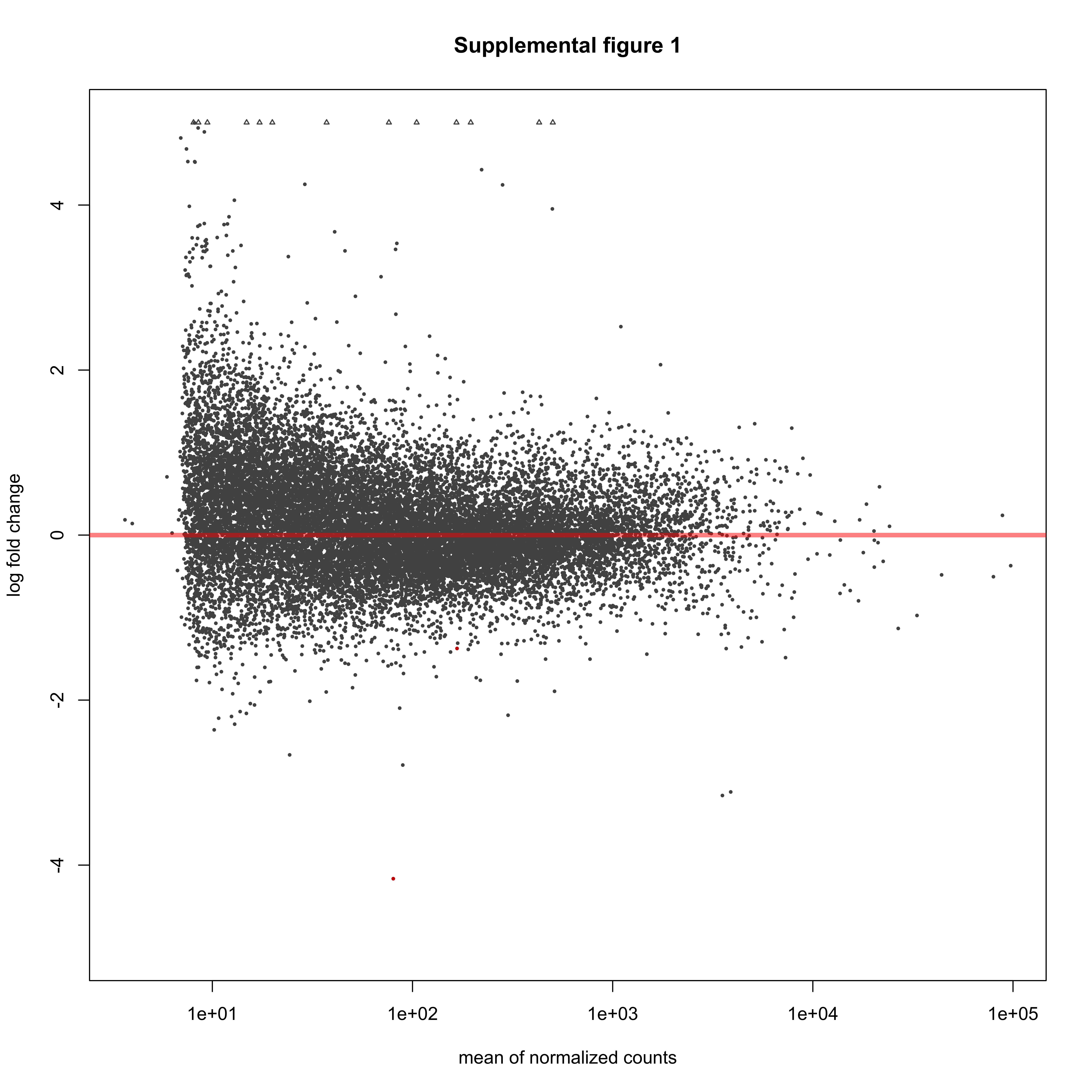

Supplement: Supplementary file 4 — Additional file 4: Figure S1. MA plot of all convalescence samples. MA plot shows the relationship of the log fold changes, plotted to the average of the normalized counts, between the convalescence samples of BM1 and EVM1 group. Each gene is represented with a dot and are colored red if the adjusted p-value is below 0.1. [file 12967_2019_2037_MOESM4_ESM.png]
