# Supplementary material for: Diagnosing enterovirus meningitis via blood transcriptomics: an alternative for lumbar puncture?
Source: J Transl Med. 2019 Aug 23;17:282. doi: 10.1186/s12967-019-2037-6 (PMC6708255; doi:10.1186/s12967-019-2037-6)
Supplement: Supplementary file 12 — Additional file 12: Additional Results. Gene expression differences between enteroviral and bacterial meningitis including the neuroborreliosis case. [file 12967_2019_2037_MOESM12_ESM.docx]

**Additional file 12: Results**

**Results: Gene expression differences between enteroviral and bacterial meningitis including the neuroborreliosis case**

The BM2 sample was excluded from the main analyses as *Borrelia burgdorferi* is a rarer cause of meningitis. If we added the BM2 sample to the comparison between the acute bacterial and viral samples, the regulation of TNF secretion GO term is not present anymore, however the specific TNF DEG is still significant. In addition, the 3 type I IFN related GO terms are still present, and even with a higher fold enrichment [response to type I IFN (12.53), type I IFN signaling pathway (13.78) and cellular response to type I IFN (13.78)] but are more mixed up with the viral GO terms (Additional file 11). Furthermore, the GO terms were completed with similar neutrophilic and leukocyte responses together with cytokines regulation and production, as we have seen without the BM2 sample.
